# Diel vertical migration into anoxic and high-*p*CO_2_ waters: acoustic and net-based krill observations in the Humboldt Current

**DOI:** 10.1038/s41598-020-73702-z

**Published:** 2020-10-14

**Authors:** Ramiro Riquelme-Bugueño, Iván Pérez-Santos, Nicolás Alegría, Cristian A. Vargas, Mauricio A. Urbina, Rubén Escribano

**Affiliations:** 1 Departamento de Zoología, Facultad de Ciencias Naturales Y Oceanográficas, Universidad de Concepción, Concepción, Chile; 2 Instituto Milenio de Oceanografía (IMO), Universidad de Concepción, Concepción, Chile; 3 Centro i~mar, Universidad de Los Lagos, Puerto Montt, Chile; 4 COPAS Sur-Austral, Universidad de Concepción, Concepción, Chile; 5 Instituto de Investigación Pesquera, Talcahuano, Chile; 6 Coastal Ecosystems and Global Environmental Change Lab (ECCA Lab), Department of Aquatic Systems, Faculty of Environmental Sciences and Center for the Study of Multiple-Drivers On Marine Socio-Ecological Systems (MUSELS), Universidad de Concepción, Concepción, Chile; 7 Departamento de Oceanografía, Facultad de Ciencias Naturales Y Oceanográficas, Universidad de Concepción, Concepción, Chile

**Keywords:** Biogeochemistry, Ocean sciences

## Abstract

A select group of marine organisms can enter the Oxygen Minimum Zones (OMZs) and even anoxic waters, while performing diel vertical migration (DVM). DVM of the euphausiid *Euphausia eximia* off northern Chile in the spring of 2015 was documented based on acoustic measurements using an echo sounder along with net samplings. Dissolved oxygen (DO) concentrations were obtained using a vertical profiler, and water samples were collected to obtain in situ nitrite (NO_2_^−^) concentrations as well as pH_T_, total alkalinity (AT), and therefore carbon dioxide partial pressure (*p*CO_2_) was estimated. Krill were found to migrate up to the surface (0–50 m) during the night and returned to ca. 200–300 m depth during the day, spending between 11 and 14 h at these layers. At the surface, DO and NO_2_^−^ concentrations were 208 and 0.14 μM respectively, while pH_T_ was 8.04 and 405 μatm *p*CO_2_. In contrast, at the deeper layers (200–300 m), DO and NO_2_^−^ were < 3 and 6.3 μM respectively, with pH_T_ 7.53 and 1490 μatm *p*CO_2_. The pH_T_ and high *p*CO_2_ values at depths represent the conditions predicted for open ocean waters in a worst-case global warming scenario by 2150. The acoustic scatter suggested that > 60% of the krill swarms enter the OMZ and anoxic waters during the daytime. These frequent migrations suggest that krill can tolerate such extreme conditions associated with anoxic and high-*p*CO_2_ waters. The inferences drawn from the observation of these migrations might have strong implications for the current oceanic carbon pump models, highlighting the need for understanding the molecular and physiological adaptations allowing these migrations.

## Introduction

Climate change is undeniably causing profound changes on Earth^1^. The consequences for life forms are far from being understood, but it is expected that the physiological diversity among species will determine a broad array of responses. Oceans are not the exemption and are likely to suffer vast climate change driven alterations. For example, the ocean acidification (OA) and carbon dioxide partial pressure (*p*CO_2_) levels, which are known to be associated with Oxygen Minimum Zone (OMZ) systems, are likely to intensify for open-ocean and coastal waters under the future scenarios of increasing atmospheric carbon dioxide release^2^. Unique biological forms that live in close association with these OMZs offer a unique opportunity to understand the adaptions required for open ocean inhabitants to overcome such projected future changes.

In the northern Humboldt Current System (NHCS), a few euphausiid species (e.g. *Euphausia mucronata*) have been described as entering to OMZs during their diel vertical migration (DVM)^3^. This OMZ (upper boundary of ∼45 µM or 1 mL dissolved oxygen L^−1^) has become one of the shallowest and most intense OMZs in the world’s oceans^4,24^, with dissolved oxygen (DO) concentrations falling below the sensor-specific detection limits (~ 3 nmol L^−1^) in a significant fraction of the OMZ waters. Such extreme oxygen-deficient waters have recently been redefined as Anoxic Marine Zones (AMZs)^4^. These AMZs have also been identified as water masses containing DO and nitrite (NO_2_^−^) concentrations ≤ 2 and ≥ 0.5 μM, respectively^5,6^.

Organisms entering the OMZs, such as euphausiids, do not only have to cope with low-oxygen water. Shallow and extensive OMZs are also recognized as areas of significant CO_2_ outgassing to the atmosphere, which is the key predictor of global warming, and a challenge for marine organisms^4,7–9^. The intrusion of sub-surface OMZs into shelf waters is already resulting in coastal acidification events, with even surface waters becoming corrosive to aragonite, as demonstrated in the eastern boundary systems of California^10^ and northern and central Chile^11–13^. The low pH and poor DO of upwelled water can reach coastal areas and readily affect the vertical distribution of marine planktonic organisms such as copepods and euphausiids^12,14^.

Despite the apparently harsh conditions in OMZ-affected areas, DVM is still a common behaviour among zooplankton species^15,16^, evolved to avoid predation and coordinated by several signals and cues, such as light, food, temperature, and salinity^15,16^. Acoustic record is a powerful tool for documenting and assessing this behaviour; enabling the detection of DVM in zooplankton swarms at high resolution, as zooplankton, euphausiids in particular, are known to avoid nets^17^. Euphausiids, like any acoustic scattering group, can be distinguished from other organisms (e.g. fishes) after proper calibration based on their acoustic properties^18,58^. For instance, acoustic observations have revealed that some organisms such as fish lack the ability to enter OMZs^60^.

Observations on zooplankton moving into and staying in AMZs during daylight hours are scarce and poorly documented, with most of the experiments conducted under controlled or simulated laboratory conditions^19,21,22^. Therefore, the primary aim of this study was to characterise the low DO/pH and high-*p*CO_2_ conditions within the AMZ in the upwelling zone off northern Chile, while reporting euphausiid migration into AMZs, using acoustic records complemented and validated with net sampling and hydrographic and chemical profilers.

## Results

### Environmental setting

**Figure 1:**
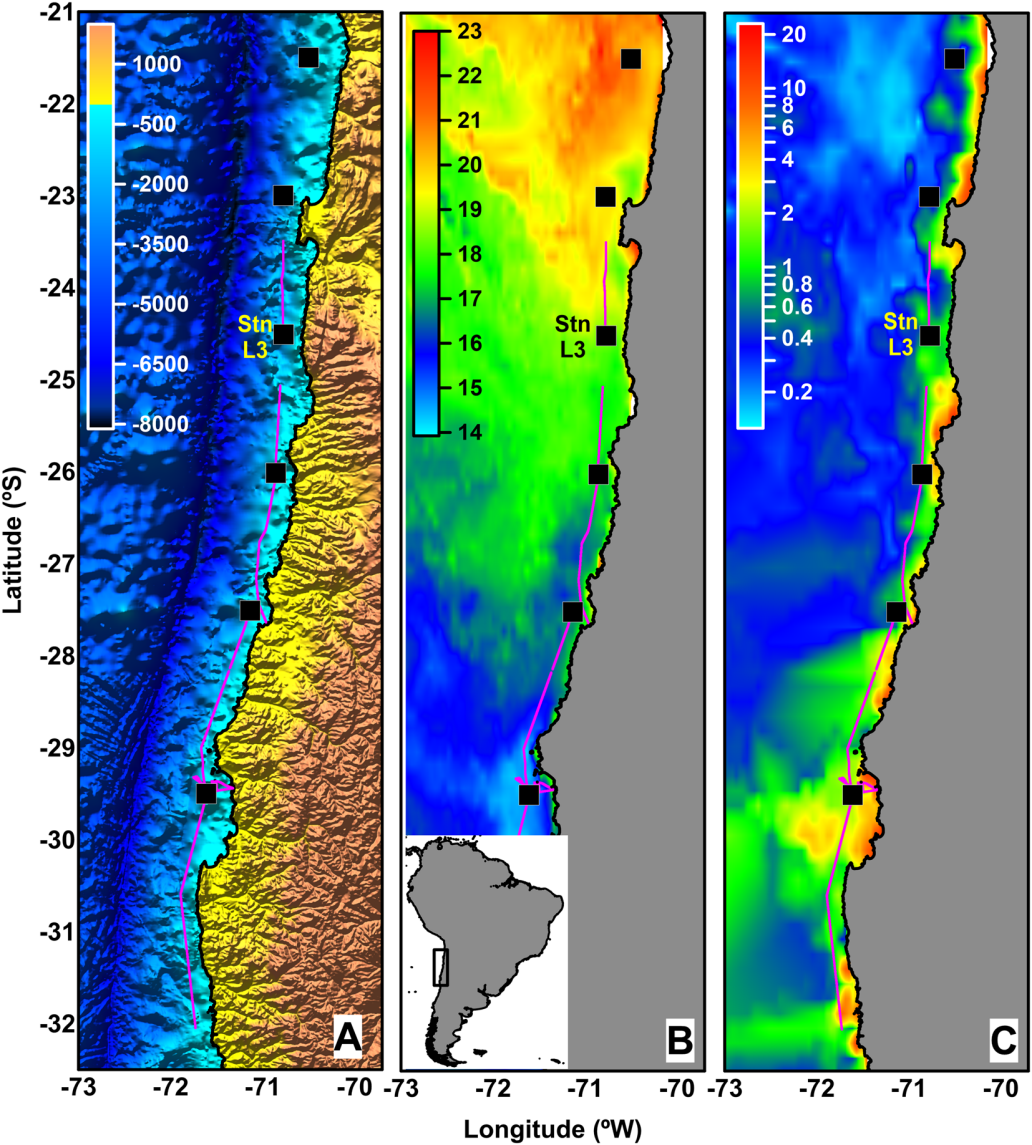
Map showing the navigation track during the LowpHox cruise in November/December 2015 off northern Chile. Black squares indicate bio-hydrographic sampling stations. Purple lines indicate acoustic measurement track. (**A**) Bathymetry (scale in m), (**B**) sea surface temperature (SST, scale in °C) and (**C**) chlorophyll-a (Chla, log-scale of mg m^−3^) maps of the study region are shown. SST and Chla maps were obtained from 8-day composite of (GOES 11/12 and Aqua-MODIS) imagery. The distributions of chlorophyll-a (chl-a) (a proxy for phytoplankton biomass) concentrations were estimated from satellite-based data during the survey period. Chl-a was estimated from 8-day composite Moderate Resolution Imaging Spectroradiometer (MODIS) Aqua Level 3 data (4-km resolution). Maps were created using Surfer software (version 16.6.484, https://www.goldensoftware.com/products/surfer).

Sampling stations were located close to the coast over areas of abrupt bathymetric changes (Stn L3, seabed ca. 1000 m depth; Fig. 1A). The oceanographic conditions in the water column changed with latitude, with solar radiation causing a slight warming at the sea surface in the northern region of the study area and colder waters in the southern region during the cruise (austral spring) (Fig. 1B). Satellite-derived surface chlorophyll-a (chl-a) concentration was higher in the southern region, most likely as a response to ongoing upwelling events (Fig. 1C).

**Figure 2:**
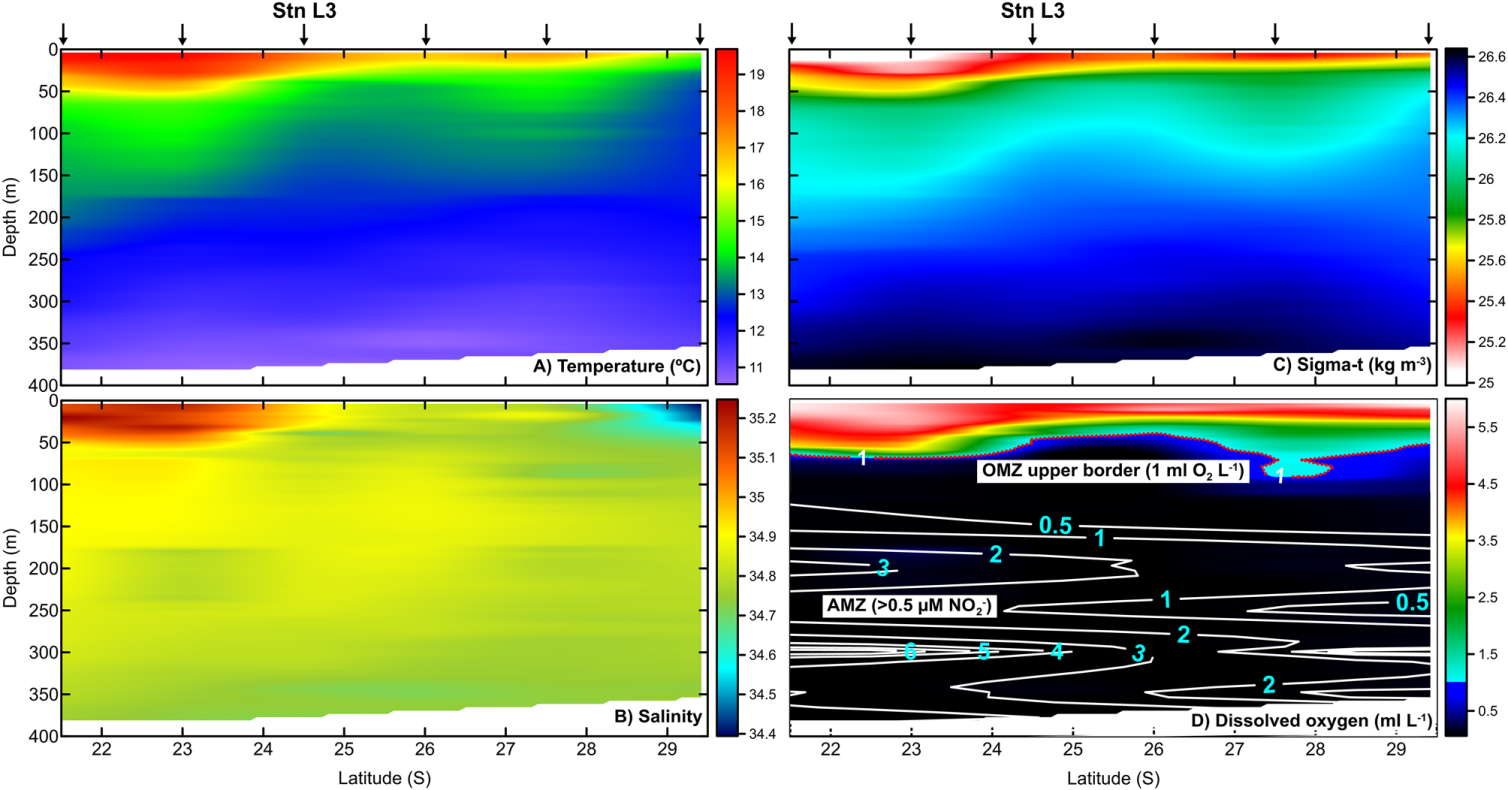
Hovmöller diagrams of water column variability recorded along longitudinal transect between December 5 and 9, 2015. CTD profiles showing temperature (°C), salinity, density (kg m^−3^) and dissolved oxygen (mL DO L^−1^), from 0 to 400 m depth along a transect from 21°30′S to 29°S. Black upper arrows indicate locations of oceanographic stations. Figure 2D: The red dotted horizontal line represents the OMZ upper limit (1 mL DO L^−1^), whereas the white isolines indicate NO_2_^−^ concentrations from discrete profiles taken along transect. Stn L3 indicates the location where hydrographic, nutrient and carbonate system profiles were selected. Plots were created using Surfer software (version 16.6.484, https://www.goldensoftware.com/products/surfer).

The continuous north–south measurements of vertical hydrographic conditions confirmed the presence of warmer waters in the northern region, whereas in the southern region cold waters almost reached the surface at 29°S latitude (Fig. 2A). Salinity was also higher in the northern region between the surface and 50 m depth, although lower salinity values were observed around 29°S, associated with freshwater inputs from the Quebrada Los Choros River (Fig. 2B). Seawater density was driven mainly by temperature, showing similar features (Fig. 2C). DO values allowed for distinguishing the OMZ down to 50 m depth, with less than 1 mL DO L^−1^ in the entire water column (Fig. 2D). The NO_2_^−^ accumulation (> 0.5 μM) indicated that the waters lack oxygen between 150 and 300 m depth and are linked to the distribution of the AMZ, extending along the coast southward and up to ca. 29°S (Fig. 2D).

**Figure 3:**
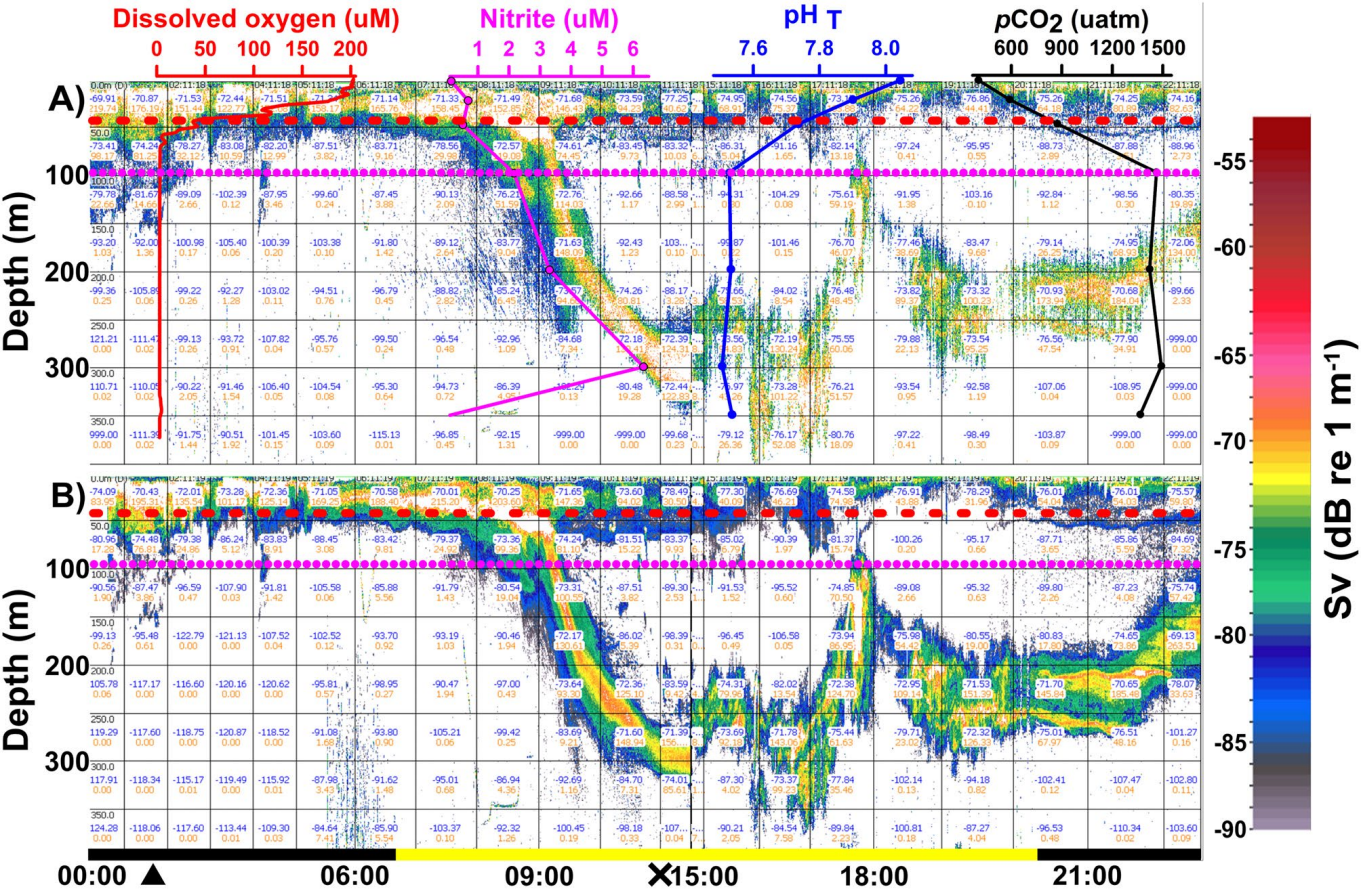
The relative backscatter measured by echo-sounder at Stn L3 during the LowpHox cruise at 38 kHz (**A**) and 70 kHz (**B**) on December 7th, 2015 (24.5°S to 26°S). Blue numbers indicate S_v_ values (dB) and red numbers indicate NASC values (m^2^ nmi^2^) for 1 h per 50 m grids. Vertical profiles selected from Stn L3 for dissolved oxygen (µM), nitrite (µM), pH_T_ (total scale) and *p*CO_2_ (µatm) were overlaid on upper panel. Horizontal red segmented and dotted purple lines indicate the Oxygen Minimum Zone upper border (defined here by 1 mL DO L^−1^ or ~ 40 μM DO) and the Anoxic Marine Zone upper border (defined here by a value of NO_2_^−^ > 0.5 μM, Thamdrup et al.^6^, Ulloa et al.^5^). Horizontal bar indicates nighttime (black) and daytime (yellow), respectively. Sunrise and sunset were 06:47 h and 20:23 h, respectively, for sampling date and location. Black triangle and cross indicate midnight and noon, respectively. Echograms were produced using Echoview software (version 9.1, https://www.echoview.com).

The vertical chemical profiles at Stn L3 in the surface layers, i.e. 0 m, showed oxygenated (OXY) waters (208 μM DO), low NO_2_^−^ concentrations (0.14 μM), pH_T_ of 8.04, and *p*CO_2_ of 405 μatm. However, a clear chemocline between 50 and 100 m depth showed an abrupt reduction in DO and pH_T_, with an increase in *p*CO_2_ levels at depths. Below 100 m and down to 400 m depth, DO was close to zero (< 3 μM). These low oxygen waters were also characterised by low pH_T_ (7.53) and high *p*CO_2_ (1490 μatm) as compared to the OXY waters, with the highest concentration of NO_2_^−^ (6.3 μM) at 300 m signalling the core of the AMZ (Fig. 3).

### Euphausiid composition

*E. eximia* conformed mono-specific layers, with no other krill species observed. Individuals were adults, mainly ovigerous females, as evidenced by observed mature gonads. Standardized abundance at Stn L3 was > 50 ind m^−3^ in the upper layer (200–0 m) during nighttime, and > 13 ind. m^−3^ in the deeper layer (200–400 m) during the day. *E. eximia* abundance was 3.8 times higher during the night than during the day.

### Vertical migration into anoxic/high-*p*CO_2_ waters

**Figure 4:**
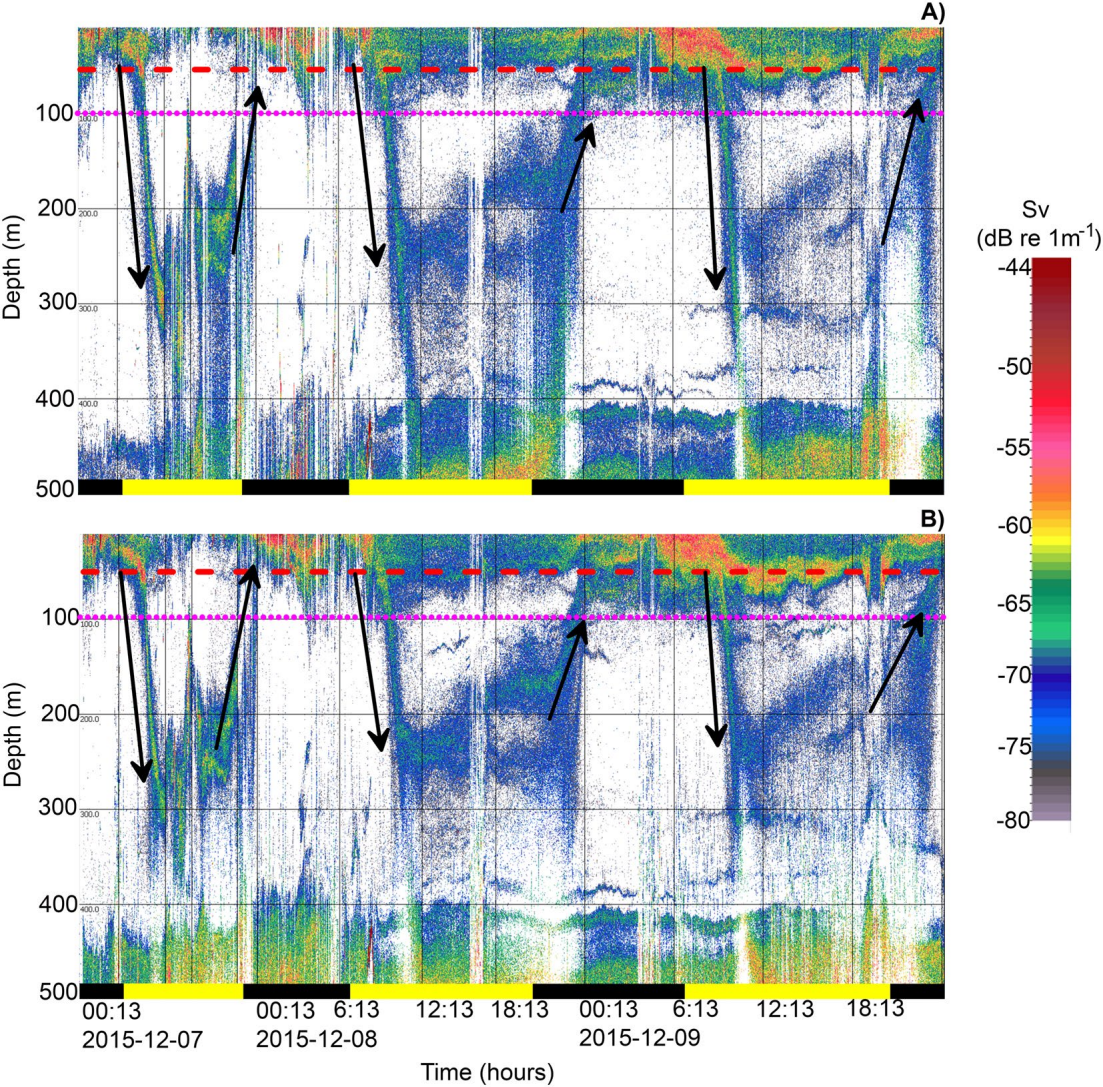
The relative backscatter measured by echo-sounder during the LowpHox cruise at 38 kHz (upper panel) and 70 kHz (lower panel) between 7 and 9th of December 2015 (23.5°S to 31.5°S). Horizontal red segmented and dotted purple lines indicate the Oxygen Minimum Zone upper border (defined here by 1 mL DO L^−1^ or ~ 40 μM O_2_) and the Anoxic Marine Zone upper border (defined here by a value of NO_2_^−^ > 0.5 μM, Thamdrup et al.^6^, Ulloa et al.^5^). Arrows indicate DVM patterns. Horizontal bars indicate nighttime (black) and daytime (yellow), respectively. Sunrise and sunset were 06:47 h and 20:23 h, respectively, for sampling date and location. Echograms were produced using Echoview software (version 9.1, https://www.echoview.com).

Algorithms applied to the datasets of 38 and 70 kHz detected high zooplankton abundance in the echograms at both frequencies along the transect. Acoustic records from both 38 and 70 kHz showed active DVM of zooplankton reaching the OMZ and even the AMZ (Figs. 3 and 4). Acoustic records along the coast (23–29°S) denoted a high eco-intensity signal, i.e. volume backscattering strength (S_v_, dB re 1 m^−1^), in the deeper layer (mostly at 200–300 m), with an S_v_ range from − 71 to − 65 dB during day, and strong acoustic signals at the surface during night (≤ 50 m). The echograms obtained on 7 December 2015 at Stn L3 provided a detailed description of DVM patterns of zooplankton (Fig. 3). After sunrise (06:47 h, local time), at 8:30 h the organisms swam down from ~ 25 m to 300 m at 11:00 h. Integrating vertical acoustic records, the swimming speed of the krill was estimated to be ca. 2.76 cm s^−1^ (or 99.28 m h^−1^) during descent. Estimated ascent speeds ranged between 0.69 and 2.06 cm s^−1^ (or 24.78 to 73.98 m h^−1^). Averaged echograms showed higher echo intensity signals (S_v_ =  − 71 to − 73 dB) and nautical area scattering coefficient (NASC, in units of m^2^ n mi^2^) (100–200 m^2^ nmi^−1^) at 175 m depth (Fig. 3).

At Stn L3, NASC values were 1.76 times higher during the day than at night, i.e. 63.7% and 36.3% respectively, indicating the high scatter signal during this time attributed mostly to *E. eximia*, and this was further confirmed by the net catches. During the day, NASC values were higher in the AMZ than in the OXY and OMZ waters, at 61.1%, 31.5%, and 7.4% respectively (Table 1). The NASC values for these layers were statistically different (PERMANOVA test, p < 0.001). The layer considered, i.e. OXY, OMZ and AMZ, was the most significant factor explaining the differences in NASC values. However, significant interaction between these factors was found when considering the time period along with the oxygen conditions (Table 2).

**Table 1:** Relative krill abundance as Nautical Area Scatter Coefficient (NASC m^2^ nmi^−1^) in relation to time and oxygen condition at Stn L3 during Lowphox cruise, December 7, 2015. OXY, oxygenated layer (> 1 mL O_2_ L^−1^); OMZ, oxygen minimum zone (< 1 mL DO L^−1^); and AMZ, anoxic marine zone (> 0.5 µM NO_2_^−^). LQ and UQ indicate lower and upper quartiles, respectively. Number in parenthesis = *n*.

Time	Oxygen condition	Median	LQ–UQ	Sum	%
Daytime	OXY (12)	75.1	54.3–162.0	1175.0	31.5
	OMZ (12)	9.7	3.9–21.0	275.9	7.4
	AMZ (60)	7.3	1.1–76.5	2281.4	61.1
Nighttime	OXY (9)	138.6	71.2–151.8	1093.2	51.4
	OMZ (9)	7.9	4.5–28.5	200.3	9.4
	AMZ (43)	0.6	0.1–9.3	831.5	39.1

**Table 2:** PERMANOVA test applied to the relative krill abundance as Nautical Area Scatter Coefficient (NASC m^2^ nmi^−1^) in relation to time and oxygen conditions at Stn L3 during Lowphox cruise, December 7, 2015. OXY, oxygenated layer (> 1 mL DO L^−1^); OMZ, oxygen minimum zone (< 1 mL DO L^−1^); and AMZ, anoxic marine zone (> 0.5 µM NO_2_^−^). df, degree of freedom; SS, Sum of squares; MS, mean square; P(perm), permutation-based *p* value. *, significant value; ** very high significant value; ns, not significant value.

Source	*df*	SS	MS	Pseudo-F	P(perm)
Condition	1	228.1	228.1	0.966	0.326 ns
Layer	2	11,740	5869.9	24.853	0.001**
Condition × Layer	2	1984.7	992.33	4.2015	0.013*
Residuals	139	32,830	236.19		
Total	144	48,674			

Condition	Groups	T	P(perm)		
Daytime	OXY, OMZ	4.5662	< 0.001**		
	OXY, AMZ	3.6749	< 0.001**		
	OMZ, AMZ	0.3608	0.758 ns		
Nighttime	OXY, OMZ	5.4154	< 0.001**		
	OXY, AMZ	5.5323	< 0.001**		
	OMZ, AMZ	2.6101	0.009**		
OXY	Day, Night	1.2728	0.213 ns		
OMZ	Day, Night	0.45326	0.692 ns		
AMZ	Day, Night	4.1449	< 0.001**		

## Discussion

Acoustic scatters, confirmed by net samplings, provided clear evidence that *E. eximia* migrate to surface layers (< 50 m) at night, concentrating high abundance and biomass near the surface. The net-based night/day abundance ratio obtained was comparable to the day/night scatter ratio estimated to be 2.7 considering the AMZ layer. These acoustic observations cannot be attributed exclusively to *E. eximia* DVM. Certainly, other migrant organisms could contribute to the observed patterns; however, net catches confirmed that *E. eximia* was the dominant species in both night and day conditions. Therefore, it is plausible to assume that *E. eximia* represented mostly the DVM observed in echograms (see “Methods” section for details on acoustic processing). During the day, *E. eximia* did not avoid the depth range where anoxia and high *p*CO_2_ prevail. This special physical–chemical condition has been previously reported in other ecosystems, such as Puget Sound estuary^61^ (WA, USA), where it was found that the larval development of another krill species, *E. pacifica*, was strongly reduced at pH 7.69. Several studies have attempted to understand the effects of OA on krill species in different regions, mainly based on laboratory conditions. Some findings have predicted severe detrimental effects on embryonic development, larvae and post-larvae growth, reproduction, fitness, behaviour, metabolism, survival and inter-moult period^21,30–32,62^, while others suggest the capacity of these organisms to tolerate high CO_2_ waters^33,34^. However, all these studies have only manipulated CO_2_ levels under normoxic (air-saturated) conditions and, therefore, without truly representing natural OMZ and AMZ conditions. It has been suggested that the efficiency of the aerobic respiration is dependent on the ratio *p*O_2_/*p*CO_2_^63^, pointing out that the effects of low DO in some oceanic areas could be exacerbated by the increase in CO_2_. The physiological basis for the arguments by Brewer and Peltzer^63^ comes from the fact that haemoglobin has an optimal pH for carrying oxygen (O_2_)^35^. Further, Mayol et al.^36^ demonstrated that high *p*CO_2_ further challenges respiration in the NHCS. However, recent evidence in fish shows that they are better adapted to tolerate hypoxia under elevated CO_2_ conditions as compared to hypoxia alone^64^. Certainly, the effects of *p*CO_2_ and *p*O_2_ cannot be considered independently, as they co-occur in nature and are both involved in respiratory processes. Although there is no consensus on mechanisms nor on the interactive effects of simultaneous changes of O_2_ and CO_2_ on animal metabolism, it is clear that *E. eximia* has adapted to survive up to 14 h in these conditions.

When integrating information from Figs. 3 and 4, it became clear that the *E. eximia* individuals were capable of crossing the OMZ and reaching the AMZ where anoxic/high-*p*CO_2_ waters prevailed. They tolerated this condition for at least 11–14 h during their DVM activity. Some species of zooplankton, such as *E. eximia*, are clearly adapted to inhabit OMZs during the day^3,22,29^. For instance, Tremblay et al.^70^ suggested that some Humboldt krill species are able to depress their metabolism based on a regulation index. Our observations indicated that *E. eximia* can tolerate the AMZ in the NHCS for extended periods. During daytime, krill should have little aerobic scope in the OMZ and AMZ; since environmental DO partial pressures (*p*O_2_) are below DO critical partial pressure (P_crit_) (i.e. *p*O_2_ < P_crit_) to sustain aerobic metabolism. Therefore, our observations suggest that *E. eximia*´s metabolism is supressed or maintained at its minimum, perhaps fuelled by anaerobic pathways. In their study, Seibel et al.^22^ found that *E. eximia* exhibited metabolic suppression during DMV, tolerating a *p*O_2_ of 0.8 kPa (or ∼15 µM DO at 10 °C) for at least 12 h. Our observations and estimations agree with this previous report for *E. eximia,* although the acoustic records and NO_2_^−^ measurements at Stn L3 showed that this species can even stay between 11–14 h in anoxic waters. This confirms the ability of *E. eximia* to suppress their metabolism, which might be more advantageous in the long term, than an enhanced anaerobic metabolism, which might still play a crucial role in short term.

From the DO vertical profile, a *p*O_2_ of 0.23 kPa (or ∼3 µM DO at 11.7 °C) was estimated at the AMZ core (300 m). This *p*O_2_ value could be even lower or 0, according to NO_2_^−^ measurements (as anoxia proxy), which would make this krill species the first to be reported to temporarily enter such extreme natural conditions. Kiko et al.^65^ previously reported that *E. mucronata* survived several hours in anoxia at 13 °C under laboratory conditions, with a P_crit_ of 0.6 kPa at 13 °C. Oxidative stress indicators have been proposed to explain, in part, the ability of *E. eximia* to live in the OMZ. This species has shown an elevated superoxide radical production, but very low lipid peroxidation^71^. These authors^71^ suggested that *E. eximia* antioxidant enzyme activity was elevated enough to avoid oxidative damage during highly contrasting oxygen conditions during the species’ DVM^71^.

Other respiratory proxies, such as the electron transport system (ETS)^72–74^, have been reported to be significantly higher in the OMZ during the day than at night in the *E. distinguenda* in the Eastern Tropical Pacific off Mexico, supporting evidence for metabolic suppression^72^. This enhanced enzymatic activity during the day would coincide with the DVM in the OMZ. These authors suggested that higher enzyme activity would allow the organisms to tolerate environments with low oxygen in deep waters^72,73^.

The DVM speeds estimated from the acoustic scatter were comparable to those in other studies with euphausiids using a similar methodology^40^. By assuming *E. eximia* to represent mostly the DVM observed in echograms, the swimming speeds estimated for *E. eximia* ranged from 24.78 to 99.28 m h^−1^, in agreement with previous reports for *E. mucronata* (60–234 m h^−1^) ^66^. Using the relationship between swimming speed and oxygen consumption rate proposed by Torres and Childress^67^ for *E. pacifica*, we have calculated that *E. eximia* might need to consume between 0.065 to 0.098 μM O_2_ mg DW^−1^ h^−1^ during its DMV within AMZ and OMZ.

Other krill species co-inhabiting the central and NHCS, such as *E. mucronata*, have been shown to contribute significantly to the carbon fluxes by migrating into the OMZ^3^, especially through the export of carbon contained in faecal pellets. Many studies have reported that euphausiids feeding on a diatom-rich diet at the base of the oxycline may have a significant role in carbon export flux^37–39^. Acoustics-based observations should be treated with caution when determining the habitat compression or the upper limit of the OMZ, as the patterns observed could be highly species-specific, and not shared by other species.

Krill migrating into AMZ are suitable sentinels of the effects of the ongoing climate change in this “natural lab”, as this ecosystem is characterized by extreme *p*O_2_/*p*CO_2_ conditions. Further studies will be required to better understand the physiological and genetic plasticity as well as adaptability of these krill species, which allow them to survive such extreme environmental conditions. Future experiments should consider environmentally relevant exposure times, i.e. when organisms stay under extreme environmental conditions, rather than unrealistic exposure times defined a priori. The study of the *reaction norms*,^41^ described as the phenotypic expression of a single genotype across a range of environmental conditions (e.g. temperature, *p*O_2_, and/or *p*CO_2_) might be a useful experimental tool for addressing such scientific questions.

Although limited in both spatial and temporal terms, our observations during the cruise, showed that euphausiid species inhabiting the NHCS, such as *E. eximia*, migrate into anoxic and high-*p*CO_2_ waters. These water masses already have pH and *p*CO_2_ values that are predicted for open ocean waters in the future (for the year ~ 2150–2200)^1,84^ based on the trajectories of atmospheric CO_2_ release by anthropogenic activities^23^. OMZs have expanded over the past 50 years, and this expansion is predicted to continue as the climate becomes warmer worldwide^25,26^. During our research cruise, DO concentrations were close to zero within the OMZ core, where even high NO_2_^−^ concentrations established an anoxic condition^5,27^. The vertical extension of the zooplankton epipelagic community is usually limited by the presence of the OMZ^3,20,28,29,59^, but our findings not only offer acoustic confirmation of the results of previous net-based studies^42^ showing that krill migrate to a particular depth. *E. eximia* can tolerate sub-critical oxygen levels for many hours and, but also, can temporally tolerate naturally anoxic and high-*p*CO_2_ conditions. Furthermore, a large proportion of krill swarms (> 60% NASC) stayed in the AMZ, which has strong implications for carbon flux and further modelling.

## Methods

### Physical–chemical measurements

Samples were collected during the LowpHox I expedition to the OMZ off northern Chile, which was conducted in December 2015, on board the *R/V Cabo de Hornos.* Hydrographic variables were continuously measured at stations along the transect using a Pump Profiler System (PPS^27^). This equipment pumps water directly from the desired depth (at resolution 1 m) while profiling the water column with an attached conductivity-temperature-depth (CTD) system (Seabird SBE-25) that provides continuous determination of salinity, temperature, and depth. DO was measured by an oxygen sensor (Seabird SBE 43) attached to CTD. Filtered seawater was collected for nutrient analysis in plastic flasks (30 mL) and was analysed on board for NO_2_^−^. Nutrient concentrations were obtained by automated colorimetric methods^69^ using a high-resolution Auto Analyzer (SEAL Analytical, model AA3HR).

The pH samples were collected (in triplicate for each station and depth stratum) in 50 mL syringes and immediately transferred to a 25 mL thermoregulated cell at 25 ± 0.1 °C for standardisation, and pH was measured in a Metrohm pH meter equipped with a combined, double-junction, glass Ag/AgCl electrode (Metrohm model 6.0258.600). The electrode was calibrated with 8.089 Tris buffer solution at 25 °C (material supplied by Andrew Dickson, Scripps Institution of Oceanography, San Diego, USA). The pH values were reported on the total scale (pH_T_). Samples (500 mL) for alkalinity (A_T_) were poisoned with 50 µL of saturated HgCl_2_ solution, stored in borosilicate bottles, and kept in darkness at room temperature. A_T_ was determined by the open-cell titration method^43^ using an automatic Alkalinity Titrator Model AS-ALK2 Apollo SciTech. All the samples were analysed at 25 °C ± 0.1 °C. The accuracy was controlled against a certified reference material (Batch 140, A_T_ 2232.58 ± 0.80 µmol kg^−1^) and repeatability averaged 2–3 µmol kg^−1^. *p*CO_2_ estimates were performed using CO2SYS software for MS Excel^44^ set with Mehrbach solubility constants^45^ refitted by Dickson and Millero^46^. The KHSO_4_ equilibrium constant determined by Dickson^47^ was used for all calculations.

In addition, satellite-based sea surface temperature (SST) and chlorophyll-a (Chl-a) concentrations were evaluated over the study period. SST data were obtained from GOES 11/12 (5-km at coastwatch.pfeg.noaa.gov) and surface Chl-a from Aqua MODIS (L3SMI, 4-km at coastwatch.pfeg.noaa.gov). Both datasets were 8-day composites.

### Acoustic analysis

The acoustic information was obtained from the *R/V AGS 61 “Cabo de Hornos”*. This vessel is equipped with the scientific echo sounder model EK60 (Simrad, Kongsberg) with six transducers (Split-beam), previously calibrated at frequencies of 18, 38, 70, 120, 200 and 333 kHz. These transducers were installed on a retractable keel that reaches 9 m below the water surface. The echo-integration system was calibrated following the manufacturer’s specifications^49^. The calibration consisted of the insonification of a known target to correct the gain of the transducer for the estimates of target strength (TS) and volumetric backscatter force (S_v_) for the echo-integration. The target used corresponded to a copper sphere of known white strength, specific for each frequency, which was located under the vessel in the centre of the acoustic beam and outside the near field of the transducer. The system parameters during the acoustic radiation emission were set according to the CCAMLR standards (Table S1).

Acoustic data were collected during the LowpHox cruise off northern Chile (Fig. 1A). The cruise track was a coastline parallel transect with a bottom depth ranging from 800 to 1200 m. The vessel speed was between 6 and 12 knots. The full dataset covers 991 km of acoustic recording in oceanic waters from 7 December (23.5°S; 70.8°W) to 13 December 2015 (32.1°S; 71.7°W). Frequencies of 38 and 70 kHz were used to detect and separate zooplankton (euphausiids) from other dispersants in the water column. Analyses were carried out with Echoview software (version 9.1). The flow chart for the process of noise reduction is shown in Fig. S1. We excluded data from the surface (5 m depth from the transducer, i.e. “ringdown” effect) and the bottom echo. Data were filtered to remove all noise sources in an efficient and semi-automated way (user-independent), thereby avoiding the altering of acoustic measurements and biases when manually removing noise sources^76^. To identify and compensate for the attenuated pings, the “attenuated signal filter” algorithm described by Ryan et al.^77^ was used. Noise generated by interference from other acoustic equipment was removed using the impulse noise removal function^77^. Background noise was removed using the algorithm-based operator formulated by Robertis and Higginbottom^78^. Finally, the number of pings function was used to resample acoustic signals in order to better visualise daily migrations of the zooplankton swarms. The concept of noise must be understood as any acoustic signal, either biological or mechanical, and/or electrical interference not of interest or representing false measurements^48^.

We defined and limited our analysis to an effective sampling range between 5 and 400 m, given the physical limitations from near-field and sound-absorption effects related to the echo-sounder frequencies used (38 and 70 kHz). Absorption is greater for the 70 kHz frequency, which exhibits the shortest range but has a greater vertical resolution than the 38 kHz echo sounder. The 38 kHz frequencies, on the other hand, exhibit a much longer range (> 1000 m) but with limited resolution for small zooplankton scatters. However, both frequencies have proved to be efficient for studying the macro-zooplankton distributions (e.g. euphausiids)^50–53^. Díaz-Astudillo et al.^54^ used 75 kHz frequency to study the zooplankton structure and vertical migration, detecting a high abundance of macro-zooplankton groups in the fjord water of Chilean Patagonia. In this work, a maximum threshold value of − 70 dB was established^53^ to separate the zooplankton layers from other scatters (e.g. fish).

Volume backscattering strength (Sv, dB re 1 m^−1^) values from the single-frequency were re-scaled into the customary index “nautical area scattering coefficient” (NASC, in units of m^2^ n mi^2^)^55^, using a grid of 50 m (depth) by 1 h (distance). Since NASC lies in the linear domain, it can be considered proportional and suitable for indexing target abundance^56^. NASC measurements have been successfully used to estimate krill distribution and relative abundance^79–81^. Although these measurements may contain other scatterers of similar size, several studies have suggested that NASC reflects the abundance and distribution of adult krill^79,82,83^.

### Net sampling

Simultaneously with the acoustic data collection, zooplankton was collected with an opening/closing Tucker Trawl net of 1 m^2^ mouth area and 300-μm mesh. A calibrated General Oceanics flowmeter was suspended across the centre of the net mouth to estimate the volume of seawater filtered by the net. Oblique tows were from depth strata 0–200 and 200–600 m during the day as well as night. The towing speed was 2 knots per 20–40 min. The sampling device was positioned at the krill aggregation maximum scattering quantity depth, corresponding to the maximum krill individual density. The depth and scatter sensors of the ship allowed us to accurately place the sampling device in the location where the krill aggregations were detected.

Once on board, krill samples were concentrated for taxonomical analysis. A stereomicroscope was used on live krill individuals at the taxonomic level of species. *Euphausia eximia* was identified, following Baker et al.^75^, in both the daytime and nighttime samples. A random subsample was made from each catch to assess the body length, sex composition, and maturity status of the sampled individuals. *E. eximia* body lengths were measured from the front of the eye to the telson tip. The samples were then preserved in liquid nitrogen for further analysis. *E. eximia* abundance, as a representation of the entire sample, was standardised to individuals per m^3^ based on the water volume filtered by the net.

### Statistical analysis

To test the differences in the NASC values with regard to the time of day (i.e. nighttime versus daytime) and between the different oxygen conditions (i.e. OXY, OMZ, and AMZ), a distance-based permutational multivariate ANOVA analysis (PERMANOVA test, set at 9,999 permutations) was performed using PRIMER-e software (version 7.0.13)^57^. PERMANOVA is a routine for testing the simultaneous response of one or more variables to one or more factors in an analysis of variance based on a resemblance measure. Datasets were averaged for each 38 and 70 kHz paired cell and fourth root transformed before analysis. The Bray–Curtis similarity of NASC values was used as a resemblance measure among sample groups^68^. Chemical conditions, considered a categorical factor, were set according to the chemical values in the water column: OXY, > 1 mL DO L^−1^; OMZ, < 1 mL DO L^−1^; and AMZ: > 0.5 µM NO_2_^−^^5, 6^.

## Supplementary information

Supplementary Information.
