# Supplementary material for: Diel vertical migration into anoxic and high-pCO2 waters: acoustic and net-based krill observations in the Humboldt Current
Source: Sci Rep. 2020 Oct 14;10:17181. doi: 10.1038/s41598-020-73702-z (PMC7560619; doi:10.1038/s41598-020-73702-z)
Supplement: Supplementary file 1 — Supplementary Information. [file 41598_2020_73702_MOESM1_ESM.docx]

**Supplementary Information**

Diel vertical migration into anoxic and high-*p*CO_2_ waters: Acoustic and net-based krill observations in the Humboldt Current

Ramiro Riquelme-Bugueño1,2,*, Iván Pérez-Santos3,4, Nicolás Alegría5, Cristian A. Vargas2,6, Mauricio A. Urbina1,2 and Rubén Escribano2,7,+.

1 Departamento de Zoología, Facultad de Ciencias Naturales y Oceanográficas, Universidad de Concepción, Chile

2 Instituto Milenio de Oceanografía (IMO), Universidad de Concepción, Chile.

3 Centro i~mar, Universidad de Los Lagos, Puerto Montt, Chile.

4 COPAS Sur-Austral, Universidad de Concepción, Campus Concepción, Víctor Lamas 1290, Casilla 160-C, CP 4070043, Chile.

5 Instituto de Investigación Pesquera, Talcahuano, Chile.

6 Coastal Ecosystems & Global Environmental Change Lab (ECCA Lab), Department of Aquatic Systems, Faculty of Environmental Sciences & Center for the Study of Multiple-Drivers on Marine Socio-Ecological Systems (MUSELS), Universidad de Concepción, Chile.

7 Departamento de Oceanografía, Facultad de Ciencias Naturales y Oceanográficas, Universidad de Concepción, Chile

* [rriquelm@udec.cl](mailto:rriquelm@udec.cl)

Table S1. System parameters used to collect acoustic data and the calibration results.

| Parameters | Setting | |
| --- | --- | --- |
| Frequency (kHz) | 38 | 70 |
| Power setting (w) | 2000 | 750 |
| Pulse duration (ms) | 1024 | 1024 |
| Data collection range (min–max, m) | 0–1000 | 0–1000 |
| Transducer gain (dB) | 26.45 | 27.00 |
| Absorption coefficient (dB km^-1^) | 9.1 | 22.8 |
| Sound speed (m s^-1^) | 1493.9 | 1493.9 |
| s_A_ correction (dB) | -0.62 | -0.54 |


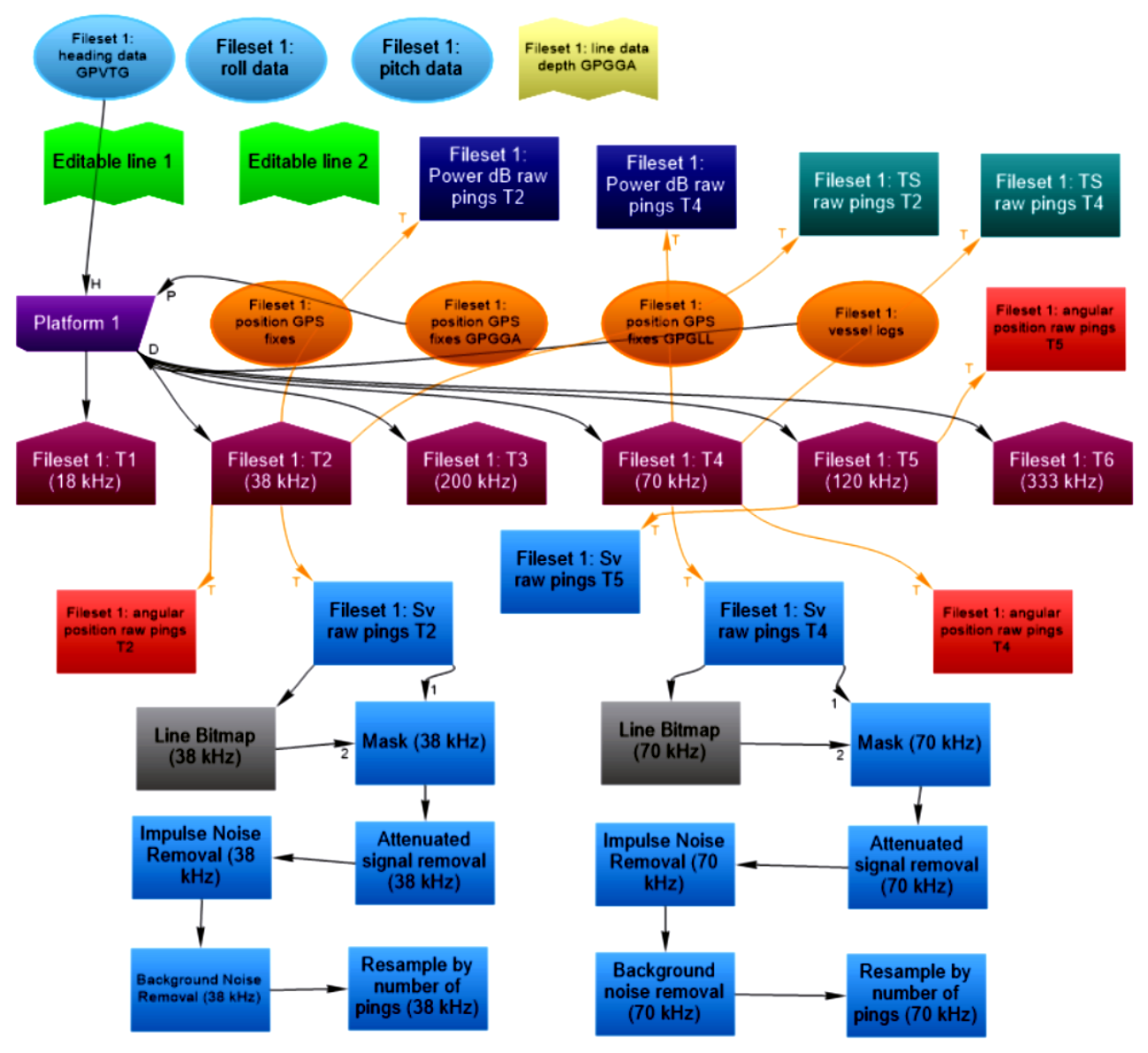


Fig S1. Flow chart (Echoview software) with operators used to analyse the acoustic data of the 38 and 70 kHz frequencies.
